# The effect of BCM guided dry weight assessment on short-term survival in Chinese hemodialysis patients: Primary results of a randomized trial – BOdy COmposition MOnitor (BOCOMO) study

**DOI:** 10.1186/s12882-020-01793-x

**Published:** 2020-04-15

**Authors:** Li Liu, Yi Sun, Yuqing Chen, Jinsheng Xu, Ping Yuan, Yulan Shen, Shan Lin, Weiming Sun, Yingchun Ma, Jianwei Ren, Wenjun Liu, Jinghong Lei, Li Zuo

**Affiliations:** 1 Renal Division, Department of Medicine, Peking University First Hospital, Beijing, China; 2 Institute of Nephrology, Peking University, Beijing, China; 3 Key Laboratory of Renal Disease, Ministry of Health of China, Beijing, China; 4 Key Laboratory of Chronic Kidney Disease Prevention and Treatment, Ministry of Education, Beijing, China; 5 Department of Nephrology, Capital Medical University Fuxing Hospital, Beijing, China; 6 Department of Nephrology, The Fourth Hospital of Hebei Medical University, Shijiazhuang, Hebei China; 7 Nephrotic Blood Purification Center, Tianjin Third Central Hospital, Tianjin, China; 8 Department of Nephrology, Beijing Miyun County Hospital, Beijing, China; 9 Department of Nephrology, General Hospital of Tianjin Medical University, Tianjin, China; 10 Department of Nephrology, Beijing Shijitan Hospital, Beijing, China; 11 Department of Nephrology, Beijing Boai Hospital, China Rehabilitation Research Center, Captain Medical University, Rehabilitation Medical College, Beijing, China; 12 Department of Nephrology, Aviation General Hospital, Beijing, China; 13 Department of Nephrology, China Academy of Chinese Medical Sciences Guang’anmen Hospital, Beijing, China; 14 Department of Nephrology, Beijing Aerospace General Hospital, Beijing, China; 15 Department of Nephrology, Peking University People’s Hospital, Beijing, China

**Keywords:** Bioimpedance, Dry weight, Hemodialysis, Mortality, Hydration status, Body composition monitor

## Abstract

**Background:**

Lack of accurate and effective assessment tools of fluid status is one of the major challenges to reach proper dry weight (DW) in chronic hemodialysis (HD) population. The aim of this randomized study was to evaluate the effect of bioimpedance guided DW assessment on long-term outcomes in Chinese HD patients. Eligible patients were randomly assigned (1:1) to two groups in each center, the control group and body composition monitor (BCM) group. In the BCM group, DW has been evaluated by bioimpedance technic every 2 months during follow-up. The primary composite endpoint consisted of death, acute myocardial infarction, cerebral infarction, cerebral hemorrhage, and peripheral vascular disease.

**Methods:**

A total of 445 patients were recruited from 11 hemodialysis centers from Beijing, Tianjin and Shijiazhuang cities from Jan 1, 2013 to Dec 31, 2014. They were randomized into either BCM group or control group. All patients have been followed up for 1 year or until Dec 31, 2014 or censoring.

**Results:**

At baseline, there were no significant differences between two groups in terms of demographic parameters, dialysis vintage, percentage of vascular access, and comorbid conditions. At the end of the study, 18 (4.04%) patients had died (11 in control group and 7 in BCM group). Kaplan-Meier survival analysis showed no significant difference in survival rates between two groups (log-rank test *P* = 0.07). However, there was an increasing trend of survival rates in BCM group compared to the control group. In the multivariable Cox analysis, there was a nonsignificant trend toward less primary composite end points in the BCM group in the adjusted analysis, the hazard ratio was impressive (0.487, 95% CI 0.217–1.091, *P* = 0.08).

**Conclusion:**

Bioimpedance technic has been applied to assess fluid status for decades and has been proved to be a promising tool for clinical practice. Although short-term outcomes were not improved in the randomized, controlled trial, the ascending trend in survival has been observed. Further studies are needed to investigate the survival benefit of bioimpedance method in DW assessment in a larger sample with longer follow-up period.

**Trial registration:**

ClinicalTrials.org, NCT01509937. Registered 13 January 2012,

## Background

Knowledge of optimal fluid status is essential in delivering care to maintenance hemodialysis (MHD) patients. Persistent overhydration (OH) causes hypertension, left ventricular hypertrophy, pulmonary edema, congestive heart failure, and leads to higher mortality [1–8]. Compared with normorhydrated patients, patients with recurrent episodes of intra-dialytic hypovolemia are at a higher risk of acute ischemic events, potentially leading to functional impairment and organ damage, including accelerated loss of residual renal function [9], blood access function loss [10], brain atrophy [11], mesenteric infarction [12], and hence, higher morbidity and mortality [13, 14].

Optimal fluid management is critical to ensure high quality of care in patients –receiving MHD. Dry weight (DW) assessment/adjustment and control of intradialytic weight gain are main components of fluid management. However, DW assessment remains a challenge in MHD patients. Gold standard measurements of fluid status, such as isotope dilution methods, are not clinically feasible due to their complexity and great expense. Inferior vena cava diameter measurements, estimations of N-terminal-pro-BNP (N-terminal-pro-brain natriuretic peptide) and other cardiac peptides have not been proved to be practical or reliable in the detection of fluid status in individual patients [15–17]. As a result, probing for DW based on trial and error has been common in clinical practice [18]. However, self-reported symptoms cannot be always reliable without knowing the medical history. Hypertension alone was found to be unreliable to define hypervolemia in part of some patients [17, 19].

Bioimpedance spectroscopy (BIS) has long been used to assess human body composition and has been extensively validated by isotope dilution methods [20, 21] and reference body composition methods [21–23]. It appears to be a promising and a valuable tool in aiding DW estimation for MHD patients. In addition, several observational studies showed the potential benefits of BIS on control of blood pressure and fluid status [1, 24]. Wizemann et al [1] found that patients who had 15% or more expansion of extra-cellular fluid (ECF) suffered higher mortality risk compared with those who had less than 15% ECF expansion estimated by BIS method in MHD patients. Recently, Onofriescu et al. found significant improvement of survival in the bioimpedance group (aiding DW by applying BIS method) compared to the clinical methods (control) group with a follow-up of 2.5 year (HR = 0.112, 95% CI, 0.014–0.918; *P* = 0.04) in a population from Turkey [25].

This multicenter, open label, single blinded, randomized controlled trial (RCT) was designed to explore the effect of BIS guided DW assessment on long-term outcomes in Chinese patients receiving MHD.

## Methods

This study design has been described previously [26]. It was approved by the Ethics Committee of Clinical Research, Peking University First Hospital and each participating center (clinical trial number: NCT01509937).

### Patients

Patients were recruited from 11 clinical sites in Beijing, Tianjin and Shijiazhuang (eight centers in Beijing, two in Tianjin and 1 in Shijiazhuang) from Jan 1, 2013 to Dec 31, 2014 [26]. Beijing, Tianjin and Shijiazhuang are three main big cities located in the middle-north part of China. Patients who were older than 18 and younger than 80 years old are eligible. Patients who initiated HD less than 3 months, been dialyzed less than five times per 2 weeks, produced urine more than 800 ml per 24 h the day before dialysis session, or a Kt/V less than 1.2 were excluded. Furthermore, we excluded patients with unstable clinical conditions (i.e. acute infection, heart failure), with pace-maker or metallic prosthesis (contraceptive device, artificial joint et al). Written informed consent was obtained from all patients.

### Study treatment

Patients were randomized to control group and Body-Composition-Monitor (BCM) group equally in each center according to the method of random number table.

Body composition and hydration state had been assessed by a portable whole body BIS device (BCM; Fresenius Medical Care, Bad Homburg, Germany). Patient measurements were obtained before the first HD session of the week. The extracellular and intracellular fluid volumes and total body water were calculated from a fluid model [20]. These fluid volumes were then used to determine the fluid overload, expressed as OH value [27]. DW_BCM_ was calculated by pre-weight minus OH (kg).

Patients in the control group received BCM measurements at the beginning and end of the study, but the results were kept blinded to the investigators. In the control group, DW was adjusted according to the dialysis center’s standard clinical practice. In the BCM group, BCM was performed at 2-monthly intervals during follow-up. In addition to routine practice, patients’ DWs were adjusted according to BCM output data following the DW adjustment strategy.

All of baseline demographics, clinical data, laboratory data, BCM measurements, ultrasonic cardiograph data and any adverse events were monitored regularly and recorded on study case report forms according to the protocol published [26].

### Study outcomes

The primary objective of the study is to compare incidence rate of the composite endpoint between BCM group and control group, composed of death, acute myocardial infarction, cerebral infarction, cerebral hemorrhage, and peripheral vascular disease. That had been judged by the committee of BOCOMO study comprised of all directors from every clinical site.

### Statistical analysis

Normally distributed variables were expressed as mean ± SD. Non-normally distributed variables were presented as median (25th, 75th percentiles). Categorical variables are presented as frequencies (percentages). Continuous variables were compared with the use of Student’s t-test or the Mann-Whitney test (for non-normally distributed data), and categorical data with the use of chi-square tests. Survival estimates and curves were generated according to the Kaplan–Meier method. Cox regression survival analysis also was performed using a backward stepwise model adjusting for demographic data (age and gender), comorbid conditions (cardiac infarction, cerebral hemorrhage, cerebral infarction and peripheral vascular disease), and other predictors (causes of end stage renal disease (ESRD), dialysis vintage, vascular access). Both Kaplan–Meier curves and Cox model used the same end point (time to event) and patients were censored when they were transferred to another dialysis center, underwent transplantation, inserted of metallic device or were still on treatment until the end of the study. All analyses were done with SAS V9.3 (SAS Institute inc, Cary, North Carolina). A *P* value of less than 0.05 was considered as statistically significant.

According to the data from Beijing Hemodialysis Quality Control and Improvement Center, the annual mortality rate of Beijing MHD patients is around 10%, it is estimated that 3 year mortality to be 30%. It is also estimated that the rate of composite endpoint within 3 year period of time is 40%. We made an assumption that BCM guided DW assessment would reduce the 3-year composite endpoint rate from 40 to 32% (20% relative risk reduction). To reach statistical significance with α < 0.05 and power > 80%, the sample size required is 1128. Allowing for a 20% loss-to-follow-up, the total sample size planned is 1354. After 1 year follow-up, 6.19% (14) of all enrolled patients (226) from 5 centers reached the composite endpoint, which was much lower than the expected rate (13.3%). Accordingly, the sample size of the study had been recalculated and 6464 patients was required to reach the statistical significance. Given the changeable difficulty to reach that number, the follow-up period of the protocol was corrected to 1 year and the study had been prematurely terminated after all patients had been followed at least 1 year.

## Results

**Fig. 1:**
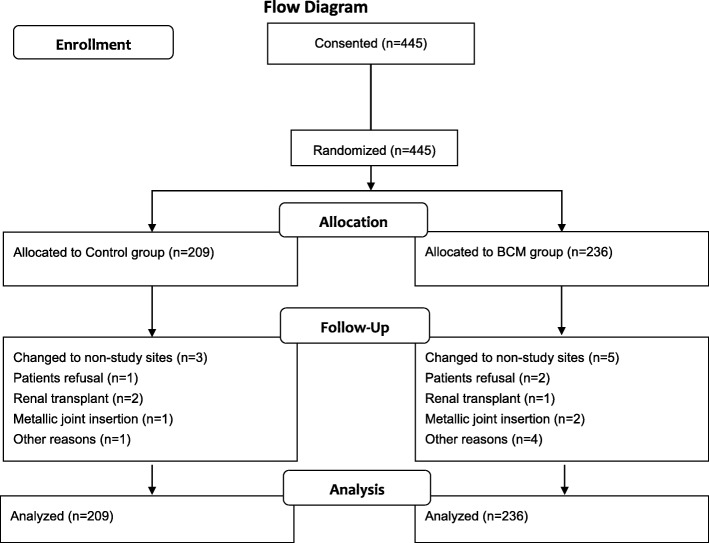
Flow diagram of the trial

A total of 445 patients were included in the final analysis (Fig. 1): 53.93% males, mean age 54.8 ± 12.7 years, median dialysis vintage of 4.13 years, 91.69% of them were dialyzed through an arterial venous fistula; approximately half of them had ESRD due to chronic glomerulonephritis (51.16%) (Table 1).

**Table 1:** Demographic characteristics and baseline laboratory parameters of the entire study subjects

	All patients (*N* = 445)	Control group (*N* = 209)	BCM group (*N* = 236)	*P*-value^#^
Age, years	54.8 ± 12.7	54.9 ± 13.3	54.7 ± 12.1	0.870
Male, N (%)	240(53.9)	109(52.2)	131(55.5)	0.479
Dialysis vintage, years	4.1(1.9, 7.5)	4.1(2.1, 7.5)	4.29(1.9, 7.5)	0.657
Follow-up, months	13.6(12.1, 15.4)	13.6(12.1, 15.3)	13.8(12.1, 15.5)	0.950
Cause of ESRD				0.663
Glomerulonephritis	220(51.2)	102(50.5)	118(51.8)	
Diabetic kidney disease	102(23.7)	53(26.2)	49(21.5)	
Hypertension	49(11.4)	21(10.4)	28(12.3)	
Others	59(13.7)	26(12.9)	33(14.5)	
Vascular access				0.387
Arterial venous fistula	408(91.7)	193(92.3)	215(91.1)	
Arterial venous graft	3(0.7)	1(0.5)	2(0.9)	
Cuffed CVC	26(5.8)	14(6.7)	12(5.1)	
Temporary CVC	3(0.7)	1(0.5)	2(0.9)	
Directive Cannulation	1(0.2)	0(0.0)	1(0.4)	
Unknown	4(0.9)	0(0.0)	4(1.7)	
History of myocardial infarction, N (%)	17(3.8)	7(3.4)	10(4.3)	0.613
History of cerebral hemorrhage, N (%)	10(2.3)	4(1.9)	6(2.6)	0.755
History of cerebral infarction, N (%)	36(8.1)	21(10.1)	15(6.4)	0.162
History of peripheral vascular disease, N (%)	9(2.0)	6(2.9)	3(1.3)	0.318
History of percutaneous coronary intervention, N (%)	6(1.4)	5(2.4)	1(0.4)	0.105
Hemoglobin (g/dl)	111.5 ± 13.7	111.7 ± 13.6	111.3 ± 13.8	0.777
Albumin (g/l)	40.8(38.4, 43.0)	41.0(38.7, 43.4)	40.7(38.2, 42.6)	0.117
Creatinine (umol/L)	927.6(787.0, 1071.0)	916.0(792.8, 1068.0)	934.5(785.9, 1075.0)	0.653
Kt/V	1.4(1.3, 1.6)	1.4(1.3, 1.6)	1.4(1.3, 1.7)	0.992
Phosphorus (mmol/L)	1.8 ± 0.5	1.8 ± 0.5	1.8 ± 0.5	0.970
iPTH (pg/ml)	209.1(90.4, 425.8)	238.5(97.0, 466.0)	191.6(83.5, 388.6)	0.090

Data are expressed as mean ± SD, median with IQR, or total number with percentages, as appropriate

#—comparison between groups

*ESRD* end stage renal disease, *iPTH* intact parathyroid hormone, *CVC* Central venous catheter

Baseline characteristics of the randomly assigned patients were listed in Table 1. At baseline, there were no significant differences between two groups in terms of demographic parameters, dialysis vintage, percentage of blood access, and comorbid conditions (myocardial infarction, cerebral hemorrhage, cerebral infarction and peripheral vascular disease).

During a median follow-up of 13.7 months, 18 (4.0%) patients died, 8 (1.8%) from cerebral hemorrhage (4 in control group and 4 in BCM group, respectively), 4 (0.9%) from infection (all in control group), 3 (0.7%) from sudden death (2 in control group and 1 in BCM group), 1 (0.2%) from heart failure (in control group), 1 (0.2%) from respiratory failure (in BCM group) and 1 (0.2%) from upper gastrointestinal bleeding (in BCM group) (Table 2). A total of 22 (4.9%) patients dropped out before the study end. The reasons for censoring included transferring to other dialysis centers (*n* = 8, 1.8%), patient refusal (*n* = 3, 0.7%), renal transplantation (*n* = 3, 0.7%), insertion of metallic joint (*n* = 3, 0.7%) and others (*n* = 5, 1.1%).

**Table 2:** list of composite primary end point by group

	BCM group(*n* = 236)	Control group(*n* = 209)	Total(N)
Death	7	11	18
Sudden death	1	2	3
Infection	0	4	4
Respiratory failure	1	0	1
Gastrointestinal bleeding	1	0	1
Heart failure	0	1	1
Cerebral hemorrhage	4	4	8
Non-fatal events	4	8	12
Acute myocardial infarction	0	2	2
Cerebral infarction	1	3	4
Cerebral hemorrhage	2	1	3
Peripheral vascular disease	1	2	3

**Fig. 2:**
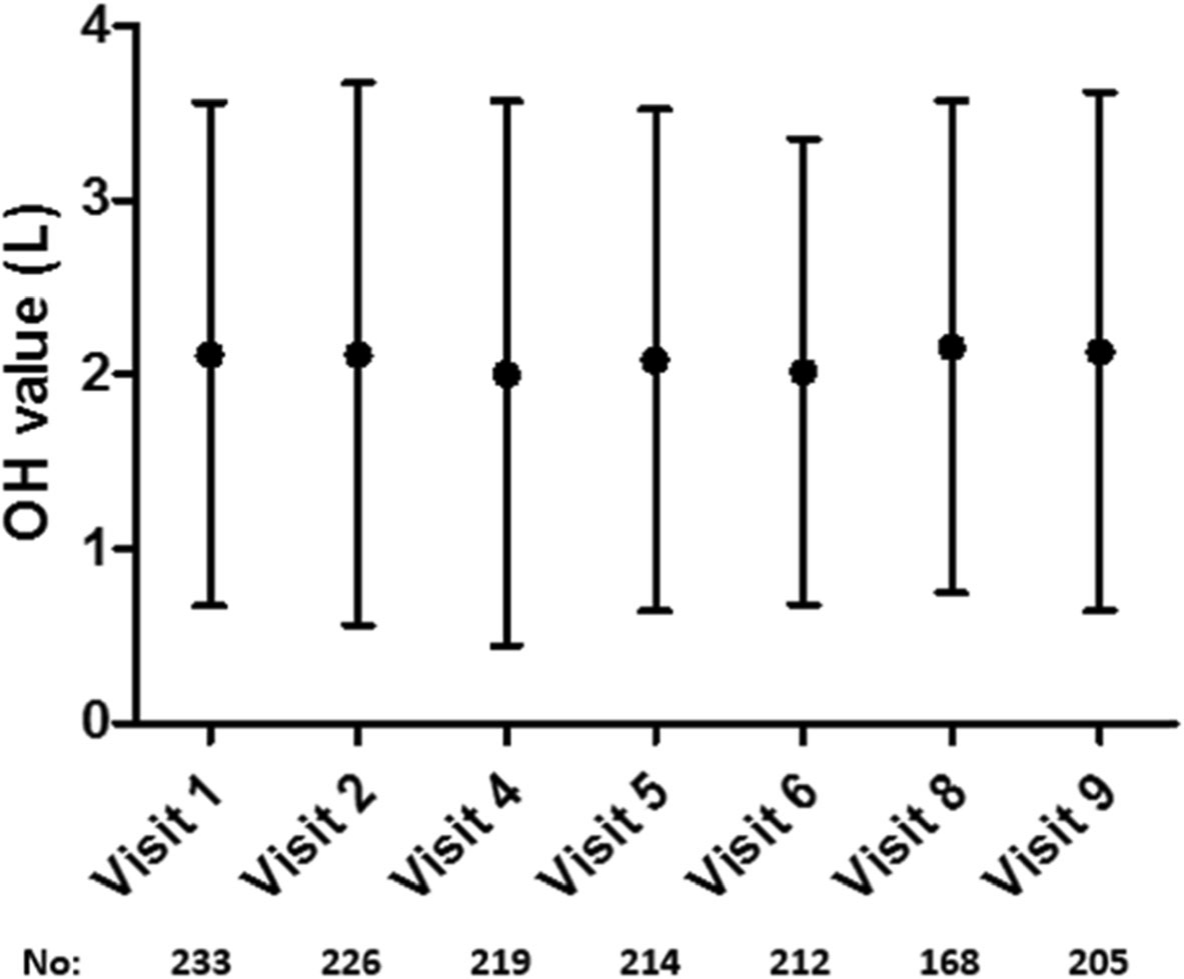
The OH values at each visit. OH: overhydration

As mentioned in the protocol, BCM measurement had been done every 2 months in the BCM group. The average of OH values at each visit in the one-year follow-up had been shown in Fig. 2 ranged from 2.11 L to 2.13 L with a standard deviation between 1.45 L and 1.49 L.

**Fig. 3:**
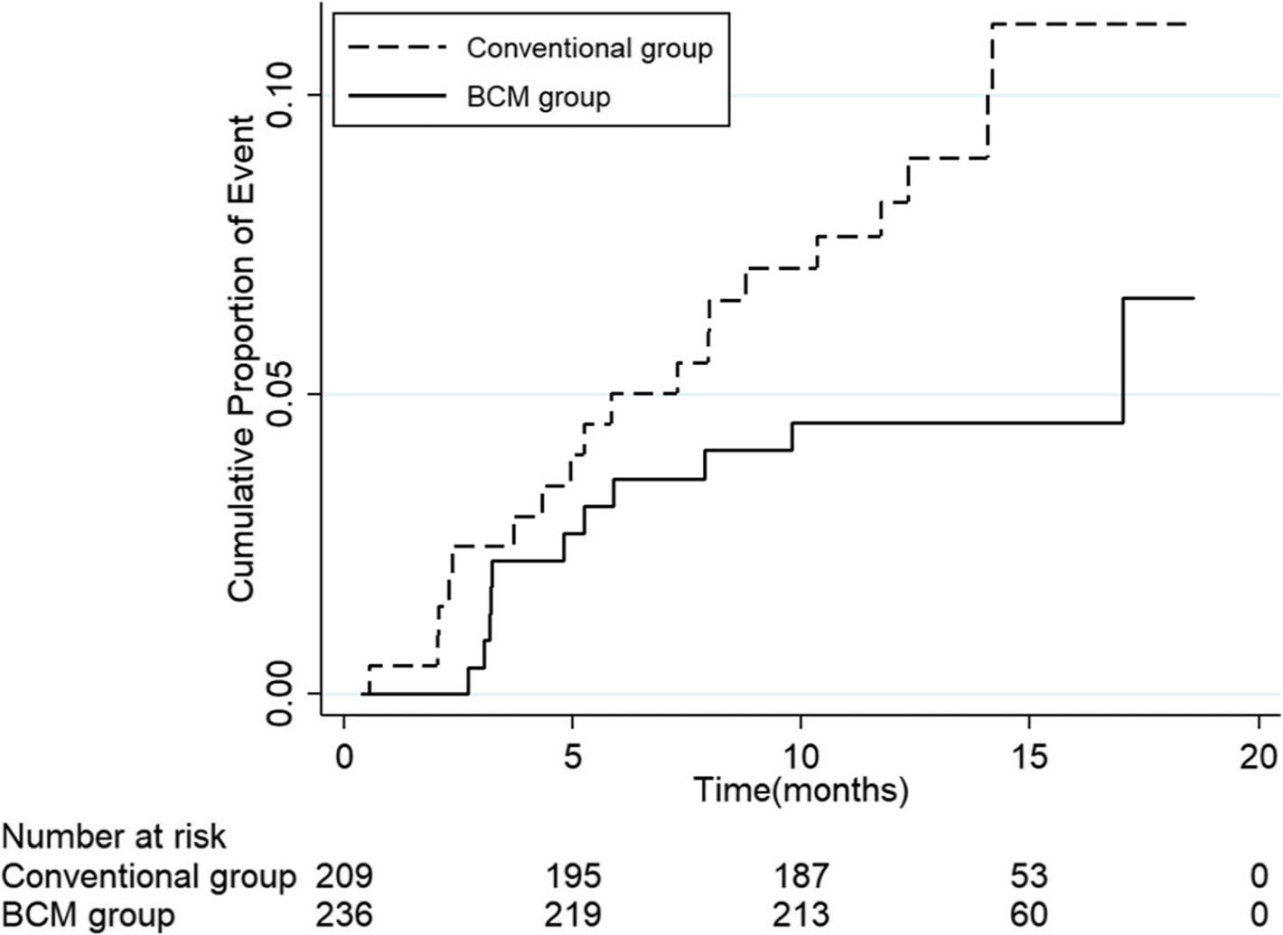
Kaplan-Meier curve comparing the survival between the BCM group and Conventional group over. BCM: body-composition-monitor. (HR = 0.51, 95% confidence interval: 0.24–1.08, log-rank test *p*-value = 0.07)

Kaplan-Meier survival analysis showed no significant difference in survival between two groups (HR = 0.51, 95% confidence interval: 0.24–1.08, log-rank test *p*-value = 0.07; Fig. 3) after 1 year follow-up. However, there was an increasing trend of survival improvement in the BCM group compared to the control group overtime.

In the multivariable Cox analysis, after adjustment for age, gender, dialysis vintage, vascular access, comorbidities and laboratory data, there was a nonsignifcant trend toward less primary composite end points in BCM group (Table 3). (HR = 0.487, 95% CI 0.217–1.091, *P* = 0.08).

**Table 3:** Results of the multivariate Cox adjusted model

Variable	Hazard ratio	95% CI		*p*-value
		Lower	Upper	
Group				
BCM group	0.487	0.217	1.091	0.0804
Control group	*Reference*			
Age	1.02	0.979	1.062	0.3379
Gender				
Female	1.173	0.451	3.051	0.7439
Male	*Reference*			
Dialysis vintage	1.039	0.947	1.14	0.4134
Cause of ESRD				
Hypertension	0.376	0.044	3.229	0.3729
Others	1.142	0.281	4.645	0.8527
Diabetes	2.533	0.948	6.765	0.0637
Chronic glomerulonephritis	*Reference*			
Vascular access				
Directive Cannulation	0	0	.	0.9968
Cuffed Central venous catheter	0.246	0.018	3.301	0.2896
Temporary central venous catheter	0	0	.	0.9951
Arteriovenous fistula	0.073	0.007	0.796	0.0318
Unknown	0	0	.	0.9944
Arterial venous graft	*Reference*			
Hemoglobin	0.993	0.966	1.022	0.6527
Kt/V	0.428	0.059	3.128	0.4033
Albumin	0.894	0.814	0.983	0.0202
Creatinine	1	0.998	1.002	0.7475
History of myocardial infarction				
Yes	0.88	0.19	4.068	0.8699
No	*Reference*			
History of cerebral hemorrhage				
Yes	1.318	0.167	10.402	0.7934
No	*Reference*			
History of cerebral infarction				
Yes	3.182	1.273	7.956	0.0133
No	*Reference*			
History of peripheral vascular disease				
Yes	3.082	0.645	14.725	0.1583
No	*Reference*			
Phosphorus	2.836	1.201	6.696	0.0174
iPTH	0.999	0.997	1.001	0.4717

*ESRD* end stage renal disease, *iPTH* intact parathyroid hormone

## Discussion

The present study was the first randomized control trial to investigate the survival benefit of BIS-based DW adjustment in the field of DW assessment in Chinese hemodialysis population. In this RCT study, although applying BCM to assess hydration status and adjust DW failed to improve the patients’ outcome compared to the clinical standard practice in 1 year follow-up, the increasing trend of survival had been observed in the BCM group.

Bioimpedance has been applied to evaluate body composition and hydration status in both hemodialysis and peritoneal dialysis patients for decades. It is not only noninvasive and easily applicable, but also well-validated [20]. It is notable that hydration status defined by BCM was associated with survival benefits had been proved by Wizemann et al. In this study, the relation between baseline hydration status and survival in 269 hemodialysis patients [1]. It showed that overhydration defined as 15% relative to the extracellular water above the normohydration target (presented an excess of ECW of 2.5 L) was linked to a more than 2-fold increased mortality risk in median follow-up of 3.5-year secondary only to the presence of diabetes.

Previous studies had demonstrated that strict BCM guided fluid management led to better blood pressure control, a decrease in arterial stiffness, a reduction of intradialytic symptoms and most importantly, a better survival [25, 28–34]. The first randomized prospective study evaluated the effect of adjustment of DW guided by BCM measurement every 3 months on survival was published by Onofriescu et al. from Turkey [25]. Normal hydration range was defined as OH value between -1 L to 1 L in this study. It was found that all-cause mortality (both unadjusted and multivariate adjusted) was significantly lower in the bioimpedance group compared to the clinical-methods group (HR = 0.1 and 0.112, respectively). Although only 131 patients were enrolled, they were followed up for a long enough period (3.5 years). On the other hand, − 2 L to 1 L after dialysis was defined as the normal target of OH value by Chen Huan-sheng from Taiwan [33]. The incidence of acute fluid overload or cardiovascular-related events decreased significantly in non-diabetes mellitus patients via monthly BCM measurement. Compared to the above target ranges of normal hydration, we used − 1.6 L to 1.6 L in our study. After 1 year follow-up with the BCM measurement in a two-month interval, although no significant results were demonstrated, the reduction trend of overall events was observed in the BCM group. In a recent systematic review and meta-analysis which included five randomized controlled trials in HD patients [35], bioimpedance-based DW assessment was proven to be associated with improvement of systolic blood pressure and of pre-dialysis fluid overload. Unfortunately, no significant improvement in survival rate was observed. Evidence for the survival benefits of BCM-guided intervention is still limited due to lack of prospective trials.

The major limitations of this trial are the insufficient number of participants and the limited follow-up period. In the previous statistical analysis, 1354 patients and 3 years follow-up were necessary to reach the statistical significance. At the end of the study, the actual rate of composite endpoint was 11.7%, lower than the predicted rate (13.3%). On the other hand, the rate of recruitment of each center is lower than expected, which induced the huge difficulties to recruit a total of 6464 patients into the study. The study was considered to be terminated earlier. Eventually, this study enrolled a total of 445 patients and had been closed after 1-year follow-up. The above factors might contribute to the insignificant result of our study. Meanwhile, the baseline characteristics demonstrated that our patients had relatively physical well-being which contributed to the better survival, for instance, lower OH value before dialysis (less than 2.5 L), both hemoglobin and albumin levels in the target range (111.5 g/L and 40.8 g/L), more arteriovenous fistula (91.7%) and shorter dialysis vintage (4.1 years). We are fully aware of the failure during the recruitment and follow-up of patients. Based on the survival trend shown in our pilot study, longer intervention time and enough sicker participants were both necessary to reach a positive result in the further study. Secondly, as an additional operation, both doctors and patients could not be kept blinded to BCM measurement in this study. Thirdly, although there were fewer patients dropped out than we assumed, more patients lost follow up in the BCM group than in the control group (14 vs. 8). The latter two both may cause bias of the results. Finally, the present study has been completed 5 years ago. We made great effort to recruit more units and patients during the following years. Unfortunately, short of finances and manpower were the main barriers.

## Conclusion

In conclusion, failure to assess hydration status is an important barrier to achieve and maintain DW in clinical practice in hemodialysis patients. Bioimpedence method has been proved to be an accurate, simple and inexpensive tool to define the DW. Given limited evidence for the survival benefits of BCM-guided intervention, it might become the basis of appropriate fluid management and reduce cardiovascular events and death, especially in sicker patients. Further well-designed studies with enough recruitment and follow-up period are necessary.

## Data Availability

The individual participant data that underlie the results reported in this article (including data dictionaries), after deidentification (text, tables, figures, and appendices) are available from the corresponding author on reasonable request.

## Abbreviations

MHD: Maintenance hemodialysis
DW: Dry weight
BIS: Bioimpedance spectroscopy
ECF: Extra-cellular fluid
BCM: Body Composition Monitor
OH: Overhydration
ESRD: End stage renal disease
iPTH: Intact parathyroid hormone
CVC: Central venous catheter
